# Pernicious Anemia Resulting in Intramedullary Hemolysis, Masking Underlying Polycythemia Vera and Mild Alpha‐Thalassemia—A Case Report

**DOI:** 10.1155/crh/8353795

**Published:** 2026-04-15

**Authors:** Amanda Rohner, Annatina Sarah Schnegg-Kaufmann, Yara Banz, Piet Rosenstock

**Affiliations:** ^1^ Department of General Internal Medicine, Inselspital, Bern University Hospital, University of Bern, Bern, Switzerland, unibe.ch; ^2^ Department of Hematology and Central Hematological Laboratory, Inselspital, Bern University Hospital, University of Bern, Bern, Switzerland, unibe.ch; ^3^ Institute of Pathology, University of Bern, Bern, Switzerland, unibe.ch; ^4^ Department of Clinical Research, University of Bern, Bern, Switzerland, unibe.ch

## Abstract

**Background:**

Vitamin B12 deficiency can cause severe intramedullary hemolysis and cytopenias. Myeloproliferative neoplasms (MPNs), particularly polycythemia vera (PV), are typically characterized by hyperproliferation but may remain undetected when masked by concomitant deficiencies.

**Case Presentation:**

A 48‐year‐old woman presented with fatigue, weight loss, and pancytopenia. Laboratory tests showed severe macrocytic anemia, hemolysis, and markedly reduced vitamin B12 levels. Gastroscopy and antibody testing confirmed autoimmune atrophic gastritis with pernicious anemia. Bone marrow biopsy revealed hypercellularity, panmyelosis, and myelofibrosis (MF‐2), initially interpreted as reactive. After vitamin B12 supplementation, cytopenias resolved; however, follow‐up demonstrated polycythemia, leukocytosis, and thrombocytosis. Molecular analysis identified a JAK2 V617F mutation (variant allele fraction 40%), confirming PV with progression toward myelofibrosis. The patient was treated with phlebotomy, low‐dose aspirin, and hydroxyurea, alongside continued vitamin B12 replacement. In addition, mild alpha‐thalassemia was diagnosed in the course of an increasingly microcytic hypochromic blood count.

**Conclusion:**

Severe vitamin B12 deficiency may mask an underlying MPN, as well as other hematologic disorders like alpha‐thalassemia. Close follow‐up after hematologic recovery is essential to avoid delayed diagnosis of coexisting hematologic malignancies and disorders. This case presents a combination of complex and extremely rare hematological scenarios, where various hematological disorders exert conflicting influences on red blood cell indices, making diagnosis challenging.

## 1. Introduction

In Western populations, the prevalence of vitamin B12 deficiency ranges from 3% to 6%, increasing with age [1]. Both vitamin B12 and folic acid deficiencies can disrupt hematopoiesis through similar pathophysiological mechanisms. Histomorphologically, these deficiencies often result in megaloblastic changes in all nucleated precursor cells in the bone marrow.

In severe cases, vitamin B12 deficiency can present as intramedullary hemolysis, mimicking thrombotic microangiopathies (TMAs), such as thrombotic thrombocytopenic purpura or hemolytic uremic syndrome [2, 3]. Peripheral blood analysis reveals signs of hemolysis, as evidenced by elevated indirect bilirubin levels, increased LDH, and decreased haptoglobin concentrations. However, anemia due to vitamin B12 or folate deficiency is usually hyporegenerative with low reticulocyte counts, which distinguishes it from TMA and other forms of peripheral hemolytic anemias [4, 5]. Less well‐known features include circulating red cell precursors, splenomegaly, and hypercellular bone marrow with increased erythropoiesis [5].

Vitamin B12 deficiency can result from a variety of causes, including inadequate intake (e.g., in cases of veganism), chronic alcohol abuse, and age‐related factors. It can also arise from conditions such as autoimmune gastritis (pernicious anemia [PA]) and other malabsorption syndromes after surgery including gastrectomy or ileum resection [6].

Myeloproliferative neoplasms (MPNs) are a group of malignancies of the hematopoietic stem cell characterized primarily by exaggerated proliferation [7]. In addition, inflammatory changes of the bone marrow can lead to myelofibrosis, and all MPNs carry a risk of progression to acute myeloid leukemia (AML). Among MPN, two groups are distinguished: The first is chronic myeloid leukemia (CML), which is driven by the t(9:22) translocation (Philadelphia chromosome), and the second group contains the Philadelphia‐negative MPNs polycythemia vera (PV), essential thrombocytosis (ET), and (prefibrotic) primary myelofibrosis (PMF) [7]. We will subsequently focus on this second group. The most frequent oncogenic driver mutations in Philadelphia‐negative MPNs are the Janus kinase 2 (*JAK2*) V617F mutation, followed by mutations of *CALR* (encoding for calreticulin), the thrombopoietin receptor gene (*MPL*) and other *JAK2* mutations. The *JAK2 V617F* mutation leads to constitutional activation of the JAK‐STAT pathway and growth factor–independent proliferation. The clinical presentation of MPNs is highly diverse and can range from asymptomatic cytosis (polycythemia, leukocytosis, and/or thrombocytosis), venous and arterial thromboembolism (including in particular mesenteric thrombosis), constitutional symptoms, and typically for PV, aquagenic pruritus and erythromelalgia. Furthermore, patients may present with hepato‐/splenomegaly and abdominal discomfort. Diagnosis is mainly based on a combination of blood values, cyto‐ and histomorphological criteria of the bone marrow, including hypercellularity of one or several lineages, and genetic criteria [7].

Searching the literature, more than 20 case reports of PV masked by PA or vitamin B12 deficiency can be found, the vast majority of which were published between the 1940s and the late 1980s, before the establishment of the current WHO diagnostic criteria for MPNs [8–13]. At the time of reporting, the neoplastic nature of MPNs was not fully understood, and in many of these historical cases, a retrospective distinction between PV and other causes of secondary erythrocytosis is not possible with certainty.

To our knowledge, this is the first documented case of PA alongside underlying PV and alpha‐thalassemia.

## 2. Case Report

A 48‐year‐old female presented to the emergency department with worsening fatigue, generalized weakness, dyspnea on exertion, and light‐headedness for several months. She also reported new, recurrent epistaxis.

The patient had lost 5 kg in the previous six months, which she attributed to reduced food intake due to stress and socioeconomic difficulties. She denied any history of bleeding disorders, gastrointestinal complaints, or blood transfusions.

Her medical history includes past gastritis and a diagnosis of Hashimoto’s thyroiditis, with a lapse in follow‐up care due to financial and healthcare access issues. Prior to presentation, she took only paracetamol and ibuprofen as needed. There was no history of tobacco, alcohol, or drug use. The family history was negative for hematological diseases.

At the time of admission, the vital signs were normal. On examination, she appeared cachectic with minimal scleritis​ and an enlarged, atrophic tongue (Figure 4 in the supporting information). No hepato‐ or splenomegaly was noted, and neurological examination was unremarkable.

Laboratory analysis showed marked pancytopenia with severe macrocytic hyperchromic anemia and hyporeticulocytosis. Furthermore, there were signs of hemolysis, accompanied by significantly elevated levels of LDH, slightly elevated bilirubin, and reduced haptoglobin. Vitamin B12 levels were severely decreased. Table 1 shows relevant laboratory findings from the time of admission until the follow‐up examination 4 months later.

**TABLE 1:** Laboratory findings from initial presentation to follow‐up 4 month later.

Parameter	Initial presentation	Follow‐up 4 months later	Reference range
White blood cells (WBCs)	2.86 × 10^3^/µL	11.36 × 10^9^/L (H)	4.0–10.0 × 10^3^/µL
Hemoglobin	39 g/L (L)	163 g/L (H)	120–160 g/L
Hematocrit	0.12 (L)	0.55 (H)	0.36–0.47
Erythrocytes	1.25 × 10^6^/µL (L)	7.29 × 10^12^/L (H)	4.0–5.2 × 10^6^/µL
MCV	98 fL	75 fL (L)	80–96 fL
MCH	31 pg	22 pg (L)	27–33 pg
MCHC	317 g/L (L)	298 g/L (L)	320–360 g/L
Platelets	81 × 10^3^/µL (L)	568 × 10^9^/L (H)	150–400 × 10^3^/µL
Reticulocytes (absolute)	14 G/L (L)	93 G/L	25–85 G/L
Reticulocytes (%)	1.16%	1.27%	0.5%–2.0%
Total bilirubin	21.0 µmol/L (H)	7.9 µmol/L	< 17.1 µmol/L
LDH	> 2500 U/L (H)	259 U/L (H)	< 250 U/L
C‐reactive protein	< 1 mg/L	1 mg/L	< 5 mg/L
Iron	25.2 µmol/L	5.1 µmol/L (L)	10–30 µmol/L
Transferrin saturation	53% (H)	6% (L)	16%–45%
Transferrin	1.89 g/L (L)	3.13 g/L	2.0–3.6 g/L
Ferritin (ECLIA)	Not measurable	18 µg/L	15–150 µg/L
Haptoglobin	< 0.10 g/L (L)	—	0.3–2.0 g/L
Vitamin B12	≤ 74 pmol/L (L)	≥ 1476 pmol/L (H)	140–650 pmol/L
Folic acid	10 nmol/L	32 nmol/L	> 7 nmol/L

Coombs‐negative hemolytic anemia due to severe vitamin B12 deficiency was suspected, and the patient was admitted to the hospital for further diagnostic workup. She received a total transfusion of three erythrocyte concentrates and was given daily intramuscular injections of vitamin B12 and oral supplementation with folic acid and iron. Further laboratory investigations, encompassing assessments for vitamin C deficiency, HIV, hepatitis B and C, parvovirus, and *Bartonella*, were negative (complete laboratory findings in Tables 2 and 3 from initial presentation to follow‐up 4 month later in the supporting information). Gastroscopy revealed atrophic gastritis and increased antiparietal cell antibodies were found. In summary, all findings correlated well with PA.

**FIGURE 1:**
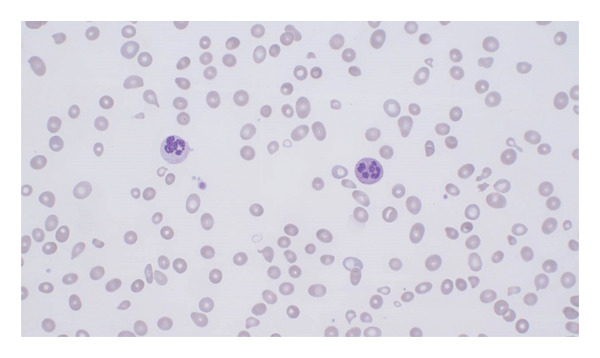
Initial blood smear: The peripheral blood smear shows marked anisopoikilocytosis with hypochromic and some oval‐shaped red blood cells, along with a few neutrophilic granulocytes.

**FIGURE 2:**
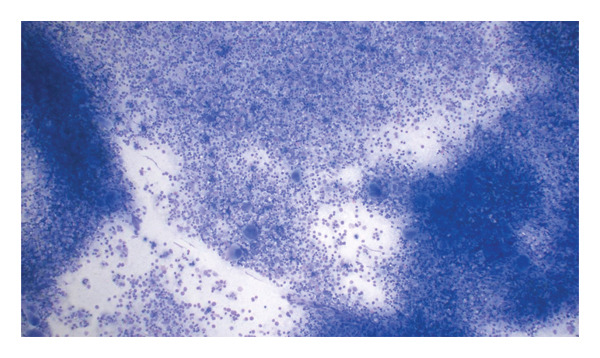
Bone marrow biopsy: The bone marrow smear shows a markedly increased overall cellularity with dense clusters of hematopoietic cells, without evidence of increased blasts.

In general, bone marrow examination is not recommended for the investigation of cytopenias due to vitamin B12 or folate deficiencies. However, in this case, a bone marrow examination on the third day of vitamin B12 supplementation was performed due to the severity of the pancytopenia and the involvement of multiple cell lines (see Figure 1). The histopathological findings revealed a markedly hypercellular hematopoietic parenchyma. All three lineages were increased (panmyelosis), and megakaryopoiesis as well as myelopoiesis showed signs of abnormal maturation (see Figure 2). In addition, histopathology of the trephine biopsy revealed diffuse myelofibrosis (MF‐2).

**FIGURE 3:**
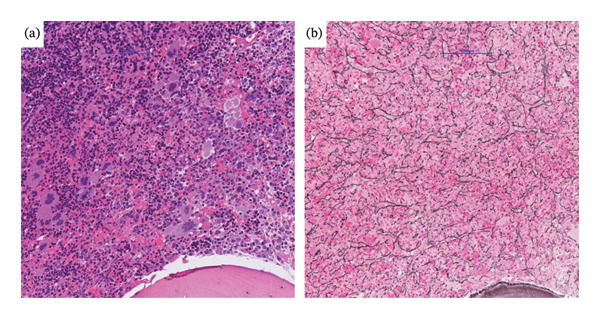
Hematoxylin & eosin stain (a) as well as silver stain (b), magnification 20x. (a) reveals markedly hypercellular bone marrow with proliferation of all three lineages. (b) reveals a diffuse myelofibrosis.

Even though the histomorphological findings were suggestive of a MPN (see Figure 3), the diagnosis of PA was retained, and the findings were interpreted as uncommon and extensive reactive changes within the context of recovering severe vitamin B12 deficiency.

In the short term, there was an adequate increase in reticulocytes, as well as a normalization of leuko‐ and thrombocytopenia, and persistent mild anemia. One month later, the patient had a near normalization of hemoglobin (11.4 g/dL, [reference: 12–16 g/dL]). The patient was also offered social and nutritional counseling and has maintained a stable body weight since.

Hematological follow‐up after four months revealed polycythemia, accompanied by thrombo‐ and leukocytosis. Erythropoietin levels were suppressed. Molecular analysis revealed a *JAK2 V617F* mutation with a variant allele fraction of 40.47%, and serum erythropoietin was 1.7 mU/mL (ref. 4.3–29.0 mU/mL). These findings prompted review of the previous bone marrow findings. Finally, we concluded that the patient most likely presented with advanced PV in transition to myelofibrosis, in accordance with the WHO 2022‐HEM5 classification. For further classification, cytogenetic studies were performed on the initially harvested bone marrow, which revealed a deletion of Chromosome X in 15 out of 20 karyotypes. A myeloid NGS panel of the same bone marrow sample showed the JAK2 V617F mutation but no additional mutations.

In parallel, therapy was initiated with low‐dose aspirin and with phlebotomy. Due to poor tolerance of the phlebotomy, cytoreductive treatment with hydroxyurea (500 mg/day) was started shortly after. During follow‐up examinations, we observed an increasingly microcytic, hypochromic blood count after adequate substitution of vitamin B12 and folic acid. Hemoglobin electrophoresis was normal, but molecular genetic testing revealed heterozygous mild alpha‐thalassemia. The patient continues to receive regular injections of vitamin B12, and other substrates are being monitored.

## 3. Discussion

We report a case of a MPN that was initially masked by severe vitamin B12 deficiency due to autoimmune atrophic gastritis in combination with low vitamin B12 intake. This case highlights several important diagnostic considerations.

Firstly, it underscores that even in patients with an underlying MPN, concomitant substrate deficiencies, particularly of vitamin B12, can lead to pancytopenia, thereby obscuring the typical hyperproliferative features of the disease. In this context, the normalization of blood counts following vitamin B12 repletion may unmask a latent myeloproliferative disorder. Clinicians should be aware that patients presenting with severe cytopenias due to nutritional deficiencies require close follow‐up to ensure complete hematologic recovery. Persistent or exaggerated responses may warrant further diagnostic workup, including molecular studies for myeloid neoplasms.

Typically, the diagnosis of PA does not necessitate bone marrow examination. However, when performed, interpretation can be challenging. In our case, several bone marrow findings such as megakaryocytic atypia and reticulin fibrosis suggested the presence of an underlying MPN but were initially interpreted as reactive changes or part of the regenerative response following correction of B12 deficiency.

It is well established that vitamin B12 deficiency can mimic various hematological malignancies, including acute leukemia and myelodysplastic syndromes. However, bone marrow fibrosis is not a characteristic feature of B12 deficiency and should rapidly prompt consideration of an alternative or coexisting diagnosis, such as an MPN.

Several cases of MPNs masked by vitamin B12 deficiency have been described in the literature, particularly in the 1960s and 1970s [8–14]. These reports led some authors to hypothesize a potential association between the two conditions. However, a case–control study by England et al. in 1968 found no increased incidence of PA among patients with PV, suggesting that the co‐occurrence may be coincidental rather than causally related [12]. We assume that in our case, PV, as well as the subsequently diagnosed mild alpha‐thalassemia, was masked for an extended period due to the vitamin B12 deficiency. The resulting anemia may also have concealed the symptoms of PV such as the persistent fatigue observed during the follow‐up period.

This is, to our knowledge, an extraordinarily rare case of PA with intramedullary hemolysis, resulting in the diagnosis of PV and mild alpha‐thalassemia during follow‐up. This case emphasizes the need for vigilance when interpreting hematological recovery after treatment of severe cytopenias. Awareness of the possibility of coexisting conditions is essential to avoid delays in the diagnosis of underlying hematologic malignancies.

## 4. Methods

Large language models (LLMs) (https://www.deepl.com; https://www.chatgpt.com) were used in the creation of this manuscript to improve readability and correct grammar.

## Funding

This research received no specific grant from any funding agency in the public, commercial, or not‐for‐profit sectors.

Open access publishing facilitated by Inselspital Universitatsspital Bern, as part of the Wiley ‐ Inselspital Universitatsspital Bern agreement via the Consortium Of Swiss Academic Libraries.

## Ethics Statement

The patient has given informed consent by signing a general informed consent form. Identifying information has not been included.

## Conflicts of Interest

The authors declare no conflicts of interest.

## Supporting Information

More results of laboratory findings are shown in Tables 2 and 3, provided in a separate supporting file. Additionally, you can find Figure 4 showing the enlarged, atrophic tongue that the patient presented with at the time of admission.

## Supporting information


**Supporting Information** Additional supporting information can be found online in the Supporting Information section.

## Data Availability

The data that supports the findings of this study are available in the supporting information of this article. Further details are available from the corresponding author upon reasonable request.
