# Supplementary material for: Pernicious Anemia Resulting in Intramedullary Hemolysis, Masking Underlying Polycythemia Vera and Mild Alpha‐Thalassemia—A Case Report
Source: Case Rep Hematol. 2026 Apr 15;2026:8353795. doi: 10.1155/crh/8353795 (PMC13081683; doi:10.1155/crh/8353795)
Supplement: Supplementary file 1 — Supporting Information Additional supporting information can be found online in the Supporting Information section. [file CRH-2026-8353795-s001.docx]

**Supplementary Material**

| Parameter | Initial Presentation | Reference Range (Adults) |
| --- | --- | --- |
| WBC (White Blood Cells) | 2.86 x 10³/µL (N) | 4.0 – 10.0 x 10³/µL |
| Hb (Hemoglobin) | 39 g/L (N) | ♀ 120 – 160 g/L |
| HCT (Hematocrit) | 0.12 (N) | ♀ 0.36 – 0.47 |
| Erythrocytes | 1.25 x 10⁶/µL (N) | ♀ 4.0 – 5.2 x 10⁶/µL |
| MCV | 98 fL | 80 – 96 fL |
| MCH | 31 pg | 27 – 33 pg |
| MCHC | 317 g/L (N) | 320 – 360 g/L |
| RDW | 36.0 % (H) | 11.5 – 14.5 % |
| PLT (Platelets) | 81 x 10³/µL (N) | 150 – 400 x 10³/µL |
| IPF (Immature Platelets) | 6.3 % (H) | 1.0 – 5.0 % |
| Normoblasts (Automated) | 2.10 % (H) | 0 % (normally absent) |
| Myelocytes | 0.01 G/L | 0 G/L |
| Metamyelocytes | 0.01 G/L | 0 G/L |
| Band Neutrophils (G/L) | 0.0 (N) | 0 – 0.1 G/L |
| Band Neutrophils (%) | 1.5 % (N) | 0 – 5 % |
| Segmented Neutrophils (G/L) | 1.4 G/L (N) | 1.5 – 6.5 G/L |
| Segmented Neutrophils (%) | 50.5 % | 50 – 70 % |
| Neutrophils (Visual, G/L) | 1.40 G/L (N) | 1.5 – 6.5 G/L |
| Eosinophils | 0.20 G/L | 0.0 – 0.4 G/L |
| Basophils | 0.00 G/L | 0.0 – 0.1 G/L |
| Monocytes | 0.0 (N) | 0.2 – 0.8 G/L |
| Lymphocytes | 1.1 G/L | 1.0 – 4.0 G/L |
| Reticulocytes (G/L) | 14 (N) | 25 – 85 G/L |
| Reticulocytes (%) | 1.16 | 0.5 – 2.0 % |
| Young Reticulocytes (IFR) | 17.3 (H) | 3 – 16 % |
| Youngest Reticulocytes (HFR) | 3.3 (H) | 0.5 – 1.5 % |
| RET-HE | 34 (H) | 28 – 35 pg |
| Bilirubin Total | 21.0 µmol/L (H) | < 17.1 µmol/L |
| C-Reactive Protein (CRP) | <1 mg/L | < 5 mg/L |
| Albumin | 40 g/L | 35 – 50 g/L |
| Iron | 25.2 µmol/L | 10 – 30 µmol/L |
| Transferrin Saturation | 53 % (H) | 16 – 45 % |
| Transferrin | 1.89 g/L (N) | 2.0 – 3.6 g/L |
| LDH | >2,500 U/L (H) | < 250 U/L |
| Direct Antiglobulin Test (DAT) | Negative | Negative |
| Ferritin (ECLIA) | See comment | ♀ 15 – 150 µg/L |
| Haptoglobin | <0.10 g/L (N) | 0.3 – 2.0 g/L |
| Vitamin B12 | ≤74.0 pmol/L (N) | 140 – 650 pmol/L |
| Folic Acid | 10 nmol/L (N) | > 7 nmol/L |
| TSH | 4.85 µU/mL (H) | 0.4 – 4.0 µU/mL |
| FT4 | 9.6 pmol/L (N) | 9 – 20 pmol/L |
| FT3 | 3.72 pmol/L | 3 – 6 pmol/L |

Table 2: The results of laboratory findings obtained at the time of admission

| Parameter | Initial presentation | Follow-up 4 months later | Reference range |
| --- | --- | --- | --- |
| WBC (white blood cells) | 2.86 ×10³/µL | 11.36 ×10⁹/L (H) | 4.0–10.0 ×10³/µL |
| Hemoglobin | 39 g/L (L) | 163 g/L (H) | 120–160 g/L |
| Hematocrit | 0.12 (L) | 0.55 (H) | 0.36–0.47 |
| Erythrocytes | 1.25 ×10⁶/µL (L) | 7.29 ×10¹²/L (H) | 4.0–5.2 ×10⁶/µL |
| MCV | 98 fL | 75 fL (L) | 80–96 fL |
| MCH | 31 pg | 22 pg (L) | 27–33 pg |
| MCHC | 317 g/L (L) | 298 g/L (L) | 320–360 g/L |
| RDW | 36.0 % (H) | 17.7 % (H) | 11.5–14.5 % |
| Platelets | 81 ×10³/µL (L) | 568 ×10⁹/L (H) | 150–400 ×10³/µL |
| IPF (immature platelet fraction) | 6.3 % (H) | 3.4 % | 1.0–5.0 % |
| Normoblasts (automated) | 2.10 % (H) | 0.00 % | normally absent |
| Myelocytes | 0.01 G/L | – | 0 G/L |
| Metamyelocytes | 0.01 G/L | 0.20 G/L | 0 G/L |
| Band neutrophils (%) | 1.5 % | – | 0–5 % |
| Segmented neutrophils (%) | 50.5 % | – | 50–70 % |
| Neutrophils (absolute) | 1.40 G/L | 7.91 G/L (H) | 1.5–6.5 G/L |
| Eosinophils | 0.20 G/L | 1.09 G/L (H) | 0.0–0.4 G/L |
| Basophils | 0.00 G/L | 0.09 G/L | 0.0–0.1 G/L |
| Monocytes | 0.00 G/L (L) | 0.57 G/L | 0.2–0.8 G/L |
| Lymphocytes | 1.10 G/L | 1.50 G/L | 1.0–4.0 G/L |
| Reticulocytes (absolute) | 14 G/L (L) | 93 G/L | 25–85 G/L |
| Reticulocytes (%) | 1.16 % | 1.27 % | 0.5–2.0 % |
| Immature reticulocyte fraction (IFR) | 17.3 % (H) | 9.9 % | 3–16 % |
| High-fluorescence reticulocytes (HFR) | 3.3 % (H) | 1.2 % | 0.5–1.5 % |
| Ret-He | 34 pg (H) | 26 pg (L) | 28–35 pg |
| Total bilirubin | 21.0 µmol/L (H) | 7.9 µmol/L | <17.1 µmol/L |
| LDH | >2,500 U/L (H) | 259 U/L (H) | <250 U/L |
| C-reactive protein | <1 mg/L | 1 mg/L | <5 mg/L |
| Albumin | 40 g/L | 40 g/L | 35–50 g/L |
| Iron | 25.2 µmol/L | 5.1 µmol/L (L) | 10–30 µmol/L |
| Transferrin saturation | 53 % (H) | 6 % (L) | 16–45 % |
| Transferrin | 1.89 g/L (L) | 3.13 g/L | 2.0–3.6 g/L |
| Ferritin (ECLIA) | See comment | 18 µg/L | 15–150 µg/L |
| Haptoglobin | <0.10 g/L (L) | – | 0.3–2.0 g/L |
| Vitamin B12 | ≤74 pmol/L (L) | ≥1,476 pmol/L (H) | 140–650 pmol/L |
| Folic acid | 10 nmol/L | 32 nmol/L | >7 nmol/L |
| Direct antiglobulin test | Negative | – | Negative |
| Erythropoietin | – | 1.7 mU/mL (L) | 4.3–29.0 mU/mL |
| JAK2 V617F | – | Detected | – |

Table 3 Laboratory findings from inital presentation to follow-up 4 month later

Abbreviations:

WBC, white blood cells; MCV, mean corpuscular volume; MCH, mean corpuscular hemoglobin; MCHC, mean corpuscular hemoglobin concentration; RDW, red cell distribution width; IPF, immature platelet fraction; IFR, immature reticulocyte fraction; HFR, high-fluorescence reticulocytes; LDH, lactate dehydrogenase; CRP, C-reactive protein; DAT, direct antiglobulin test.

(H) indicates above reference range; (L) indicates below reference range.

Reference ranges apply to adult females unless otherwise specified.


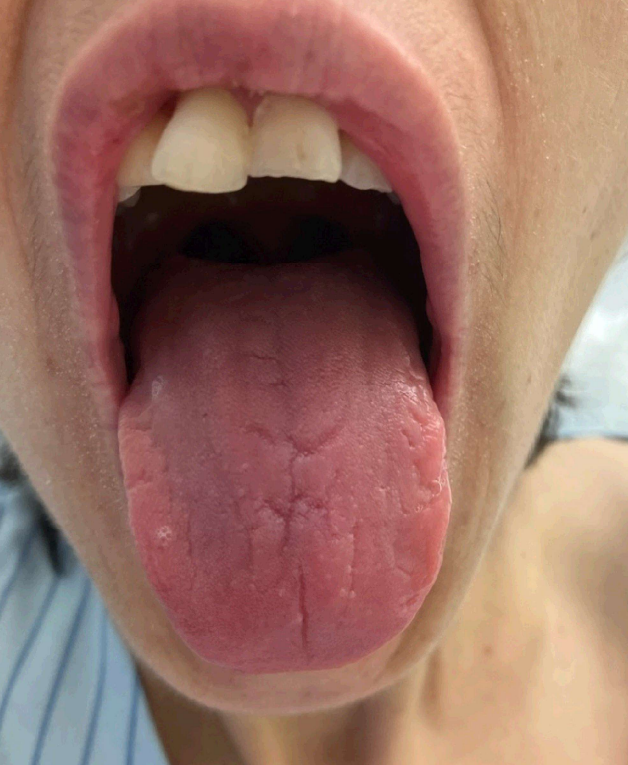


Figure 4: Hunter Glossitis, as seen above, a condition marked by a smooth, inflamed tongue, often due to nutritional deficiencies like vitamin B12.
